# Vitamin D and Osteoporosis in HIV/HCV Coinfected Patients: A Literature Review

**DOI:** 10.1155/2015/969040

**Published:** 2015-07-27

**Authors:** Paola Di Carlo, Lucia Siracusa, Giovanni Mazzola, Piero Colletti, Maurizio Soresi, Lydia Giannitrapani, Valentina Li Vecchi, Giuseppe Montalto

**Affiliations:** ^1^Department of Sciences for Health Promotion and Mother-Child Care “G. D'Alessandro”, University of Palermo, Via del Vespro 127, 90127 Palermo, Italy; ^2^Biomedical Department of Internal Medicine and Specialities, University of Palermo, Via del Vespro 141, 90127 Palermo, Italy

## Abstract

Vitamin D deficiency further increases the risk of osteoporosis in HIV-positive patients coinfected with hepatitis C virus (HCV); however, it is still unclear whether HCV-related increased fracture risk is a function of the severity of liver disease. The aim of this review was to identify studies on associative vitamin D deficiency patterns in high-risk populations such as HIV/HCV coinfected patients. We did this by searching MEDLINE and EMBASE databases, from inception to August 2014, and included bibliographies. The final 12 articles selected are homogeneous in terms of age but heterogeneous in terms of sample size, participant recruitment, and data source. Most of the HIV/HCV coinfected patients have less than adequate levels of vitamin D. After reviewing the selected articles, we concluded that vitamin D deficiency should be regarded as a continuum and that the lower limit of the ideal range is debatable. We found that vitamin D deficiency might influence liver disease progression in HIV/HCV coinfected patients. Methodological issues in evaluating vitamin D supplementation as a relatively inexpensive therapeutic option are discussed, as well as the need for future research, above all on its role in reducing the risk of HCV-related fracture by modifying liver fibrosis progression.

## 1. Introduction

Clinicians and researchers are currently using available data sets to assess the balance of beneficial and harmful effects of vitamin D not only on skeletal health but also on its potential role in nonskeletal outcomes such as cardiovascular disease, death, and quality of life [1–3].

The effects of vitamin D on immune function [4] and its immunomodulatory and anti-inflammatory properties have been recognized, and a nontraditional role of vitamin D has been reported in cancer patients and autoimmune disease [4–6].

These effects have also been reported in chronic liver disease and among chronic hepatitis C patients in whom vitamin D is involved in regulating the immune system, inflammatory response, and fibrogenesis [7–10].

Recently, low 25-Hydroxyvitamin D serum levels have been associated with the severity of liver fibrosis in genotype 1 chronic hepatitis C patients (G1CHC) [11, 12].

In vitro studies have shown that vitamin D is an antiviral agent that inhibits HCV production in a human hepatoma cell line [13]; a synergistic effect of vitamin D and interferon-alfa on HCV production has also been reported. In HCV monoinfected patients with recurrent hepatitis C after liver transplant, higher rates of virologic response were observed in those receiving vitamin D supplementation [14].

Finally, in a randomized prospective trial including only CHC treatment-naive HCV-genotype (HCV-GT) 1 patients, virologic response rates were again higher in the group receiving vitamin D supplementation [11].

Hepatitis C virus (HCV) infection has become a major health problem among the HIV-infected population [15, 16]. Approximately 30% of all human immunodeficiency virus (HIV-) positive patients are also infected with hepatitis C virus (HCV) [16–18].

Among the multifactorial mechanisms underlying skeletal disorder in the HIV and HCV setting, vitamin D deficiency is considered a risk factor for osteoporotic fracture [7, 18–20]; moreover, the finding that HCV-related increased fracture risk is a function of the severity of liver disease has generated a lot of attention so far [7, 21–23].

The fact that HIV and HIV/HCV coinfected patients are at risk of vitamin D deficiency because a wide variety of medications used to treat AIDS/HIV enhance the catabolism of 25(OH)D and 1,25(OH) 2D [23, 24] has been acknowledged. Vitamin D deficiency and bone disease in HAART patients [25] have been associated with NNRTIs [26], as well as tenofovir [27] and PIs [27, 28].

While there are recommendations for the evaluation, treatment, and prevention of vitamin D deficiency in healthy patients at risk of deficiency [3, 24] and in specific HIV populations such as young HIV-positive adults with 25-hydroxyvitamin D (25-OHD) < 20 ng/mL [29], there is still some debate about the evaluation, treatment, and prevention of vitamin D deficiency in the HIV population [30] and in particular in HIV/HCV coinfected subjects [7, 8, 31, 32].

Finally, recent literature offers recommendations on screening and treating vitamin D deficiency and osteoporosis in HIV-positive patients [30] but there is not a great deal of literature and/or consensus on cost-effective management of this patient population, especially HIV/HCV coinfected patients [22, 31–33].

This paper aims to summarize the prevalence of vitamin D deficiency in HIV/HCV coinfected patients, review available data on the association between vitamin D levels and severity of liver disease, and discuss the impact of this relatively inexpensive therapy on reducing liver fibrosis and improving sustained virologic response rate (SVR) in HCV patients.

## 2. Methods

We searched the Medline (PubMed) database for articles that matched any combination of the following keywords: vitamin D, vitamin D deficiency, 25-hydroxyvitamin D, HIV/HCV coinfections and diagnosis and treatment, and hypovitaminosis. Studies were identified through searching MEDLINE and EMBASE databases, from their inception to August 2014.

Articles were screened and those that reported on the relationship between vitamin D insufficiency/deficiency and HIV/HCV coinfections were included. We limited the search to language (i.e., Spanish, French, or English) and abstract availability. Because the terms HIV infection and HIV/HCV infection are frequently associated in the scientific literature, for this study the term HIV/HCV coinfections was used as a medical subject heading (MeSH) and the other terms (together with their linguistic variations) were used as keywords. Some articles that appeared with keyword searching were excluded because they were not relevant to the purpose of this review and tackled other topics such as anti-HCV therapy in HIV/HCV coinfected patients.

## 3. Results

Forty-four studies fit the criteria; 15 of these were duplicates and were removed. After screening titles and abstracts, we excluded 9 articles on studies involving HCV monoinfected or HIV monoinfected participants. Applying the eligibility criteria, the full texts of 12 articles were reviewed.

We selected 12 studies (see Table 1): 10 original [21, 31–39], 1 systematic review and meta-analysis article [22], and 1 review manuscript [20].

We found 5 cross-sectional [31, 32, 35, 37, 39], 3 retrospective [21, 34, 36], and 2 prospective cohort studies [33, 38]; most control groups included patients with HIV-mono- or HCV-monoinfection.

Overall, the articles were highly heterogeneous in terms of sample size, participant recruitment, and data source (Table 1). The patients' age in all the studies is relatively homogenous (median age 45 years old), reflecting the worldwide aging of the HIV population after the widespread availability of combination antiretroviral therapy (cART).

In general, the articles analyzed the prevalence of vitamin D levels in HIV/HCV coinfected patients and the association between vitamin D deficiency and liver disease variables such as severity of liver disease [21, 32, 34, 39] and the influence of vitamin D levels on virological response [32, 35, 36].

In fact, two recent studies showed a significant association between hypovitaminosis D, severity of liver disease, and response to interferon- (IFN-) based treatment in HIV-HCV patients [11, 35]. However, the association between 25(OH)D levels and SVR rates is thought to be limited to difficult-to-treat patients in whom treatment failure may depend on other factors (IL28 B, HCV genotype, hepatic expression of vitamin D receptor) [11, 12, 33, 35].

In their HIV/HCV coinfected setting, Branch et al. [36] found that baseline levels of 25(OH)D in patients treated with ritonavir are not predictors of EVR and SVR because ritonavir may influence conversion of 25(OH)D to the active metabolite. Other articles showed a significant negative association between longer duration of ART, especially PI exposure and bone mineral density (BMD) and osteoporosis [22, 28, 38]. However, in one large cohort study, HCV coinfection remained an independent predictor of osteoporotic fractures after checking for the presence of cirrhosis [21, 22].

Two articles [33, 39] showed no association between hypovitaminosis D, low BMD, and liver fibrosis (histological fibrosis staging according to METAVIR scores 0 [no fibrosis] to 4 [cirrhosis]) in HIV/HCV coinfected patients. The analysis of patient setting showed that most of the study populations included HIV/HCV coinfected African Americans. How race affects the impact of vitamin D on bone health has recently been investigated in African American men and women, revealing differences due to socioeconomic and genetic factors, such as resistance to the bone resorbing effects of PTH in the black population [33, 40–42].

Most of the patients enrolled in Milazzo et al.'s study [39] had HCV GT1 genotype and low levels of vitamin D that varied seasonally, as reported in another Italian study [9].

On the contrary, Avihingsanon et al. [32] found significant liver fibrosis in patients with HIV/HCV coinfection and low levels of 25(OH)D. These results may be influenced by race (Asian), HCV genotype (GT) [the most prevalent circulating genotype was HCV GT3 (47%)], and IL28B polymorphism (the major allele [CC genotype] of rs12979860 position was found in 88% of HIV/HCV patients).

Regarding the prevalence of fracture in this group of patients with liver disease, Dong et al.'s systematic review and meta-analysis [22] highlights that fracture incidence rate ratios (IRRs) are higher for coinfected than HIV monoinfected and uninfected patients. In multivariate analyses of HIV/HCV coinfected individuals, older age, lower BMI, postmenopausal status, and time on protease inhibitor were significantly associated with osteoporosis [18–20, 22, 27, 28, 38].

Among the HIV/HCV coinfected patients, haemophiliacs are considered to be at the highest risk for fracture. Table 1 includes Linari et al.'s study [37] on the prevalence of osteoporosis in this group of patients. The authors divided 78 haemophiliac patients into three groups (uninfected, HIV monoinfected, and HIV/HCV coinfected); hypovitaminosis D and low BMD were present in all patients, with lower L-DXA scores in coinfected patients and a more evident increase of bone resorption markers in HIV and HIV/HCV coinfected patients.

## 4. Discussion

Our literature review indicated that vitamin D deficiency is common in HIV/HCV coinfected patients and that hypovitaminosis D occurrence among patients with an average age of 45 years should raise concerns about the risk of developing bone fractures.

Low levels of vitamin D were also found in HIV monoinfected [21, 38, 39] and HCV monoinfected control groups [32, 37].

However, the selected articles reveal some aspects that prompt us to reconsider the definition of hypovitaminosis D. In fact, the significance of vitamin D deficiency has several limitations because 25-hydroxyvitamin D concentrations varied by age, season, study sample size, and methodological assay approach [25(OH)D assay used] [37, 39, 40]. Moreover, black and Hispanic individuals synthesize less vitamin D per unit of sun exposure than white individuals [33, 41, 42]. Therefore, levels of vitamin D in HIV/HCV coinfected patients should be monitored according to the reference range for the sample setting. In fact, further validation of the reported results is needed, since studies conducted on larger cohorts, as well as in Italian coinfected patients [39], have revealed low vitamin D levels consistent with those recently reported for healthy populations from Western countries [38, 39, 43].

Milazzo et al. found that season and severity of fibrosis were predictors of low 25(OH)D in an Italian HIV/HCV coinfected population and that median 25(OH)D serum levels below 25 ng/mL were similar in the HIV monoinfected, HIV/HCV coinfected, and healthy controls [39]. In a recent, large study on a general healthy population in Central Europe, Pludowski et al. [43] reported an average concentration of less than 30 ng/mL of 25(OH)D.

Guidelines specify that 25(OH)D concentrations are the best indicator of overall vitamin D status in the general population and define vitamin D insufficiency as a 25(OH)D level of below 75 nmol/L (30 ng/mL) and deficiency as below 50 nmol/L (20 ng/mL) [40]. However, the Endocrine Society Clinical Practice Guidelines (ESCPG) suggest that the vitamin D requirements of sick patients may be greater than those of healthy individuals, and blood levels above 30 ng/mL may carry additional health benefits by reducing the risk of various disease conditions [24, 40].

The Hormone Foundation's Patient Guide to Vitamin D Deficiency suggests that patients with chronic (long-term) liver disease are at high risk of deficiency; therefore vitamin D testing is recommended and patients should be given advice about adequate dietary intake and medical supplementation to prevent and treat deficiency [44].

The influence of diet on vitamin D status is minimal (accounting for 3.7–5.9 *μ*g or 148–236 IU daily) as only a few foods, such as sardines, tuna, and mushrooms, naturally contain vitamin D [24, 40]. Today, the main sources are foods which have been fortified with vitamin D2 and/or vitamin D3, such as milk, orange juice, yoghurt, cheese, and breakfast cereals, which people of all ages can include in their daily diet. Although it has been suggested that a Mediterranean-style diet or a diet rich in fish and other foods containing vitamin D has health benefits, further evaluation is necessary [24, 38, 44, 45].

Our selected articles investigated the possibility of an association between vitamin D deficiency and hepatic fibrosis in HIV/HCV coinfected patients. One particular study that involved mostly African American HIV–HCV coinfected patients showed that increasing vitamin D levels does not improve bone or liver outcome [33], whereas other studies illustrate how vitamin D influences virological response to antiviral treatment in HIV-HCV coinfected patients and has a role in liver fibrosis progression [31, 34, 35].

Older and more recent research has investigated the link between vitamin D homeostasis and bone loss in patients with liver disease [7, 8, 22, 46]. Vitamin D deficiency in chronic liver disease is only partly the result of a synthesis dysfunction of the liver or/and decreased vitamin D absorption caused by intestinal edema due to portal hypertension or to cholestasis.

Recently, significantly lower levels of 25-hydroxyvitamin D were observed in patients with liver cirrhosis, admitted for acute decompensation, suggesting that systemic inflammation or liver dysfunction has an impact on 25(OH)D level [46].

Regarding vitamin D synthesis, parathyroid hormone (PTH) is involved in its activity and expression; disturbance of the parathyroid hormone-vitamin D axis with bone mass loss in chronic liver disease has recently been reported in cirrhotic postmenopausal women and in geriatric patients with vitamin D deficiency [47, 48].

A systematic review and meta-analysis included in our literature review found that HIV/HCV-coinfected patients are at higher risk for osteoporosis and fractures than HIV-monoinfected controls and are at a substantially higher risk than uninfected controls. HCV and viral hepatitis coinfection remained an independent predictor of osteoporosis [22].

HCV and HIV infections are both associated with increased levels of proinflammatory cytokines that can promote osteoclastogenesis or inhibit osteoblast differentiation and collagen synthesis [7, 8, 22].

The mechanisms responsible for osteopenia and osteoporosis are uncertain and multifactorial, but exposure to certain antiretroviral drugs (in particular a NRTI: tenofovir-TDF-and the PI class), aging, HIV itself, parathormone (PTH) increase, and vitamin D deficiency may be implicated.

Moreover, in a cohort of G1CHC patients, the hepatic expression of VDR protein is associated with severity of both liver fibrosis and inflammation [12, 34].

Guidelines for the management of osteoporosis in HIV-negative [3, 24, 40, 44] and HIV-positive patients identified adequate vitamin D status, in addition to calcium from diet or supplements, as essential for the prevention of osteoporosis [24, 29, 30, 49]. Adjunct therapy with high-dose, daily vitD3 for HIV-infected subjects and for those on/off highly active antiretroviral therapy was recently investigated in a high-risk, adult HIV-infected group and in HIV-infected children [29, 49]. However, these levels have not been supported by adequate dose-finding RTC studies.

Our review highlights important areas to explore for future prevention strategies. Future interferon-free direct-acting agents may have a better effect on bone metabolism and decrease fracture incidence after successful treatment. Although the risk of fracture is clearly higher in HIV/HCV coinfected individuals, it is not clear if DXA screening of these individuals before the age of 50 is a cost-effective prevention method and requires further study.

Lifestyle-related factors appear to have a substantial impact on the risk of fractures in HIV/HCV coinfected individuals but, based upon available studies, this cannot solely be attributed to alcohol and substance use [22, 38].

Recent data indicate that vitamin D supplementation is a relatively inexpensive therapeutic option to reduce liver fibrosis and improve SVR [22, 34]. Interestingly, two potentially modifiable factors, CD4+ nadir and serum 25(OH)D levels, were both independent modulators of liver fibrosis progression and determinants of portal pressure [34].

Currently, practitioners are often concerned about the lack of well-characterized data on the therapeutic value of vitamin D supplementation to reduce liver fibrosis progression in HIV/HCV coinfected patients. Increasing vitamin D intake may positively modulate response to antiviral treatment in HCV-infected or HIV/HCV coinfected patients, and, in association with standardized treatment for chronic liver disease, it could be of benefit in reducing liver fibrosis progression in HIV/HCV coinfected patients.

In general, there is no evidence to suggest that increasing the recommended vitamin D intake for the general population to 20–50 *μ*g (800–2000 IU) would cause any medical problems. However, the authors recommend careful clinical observation and laboratory monitoring when higher doses of vitamin D supplements are administered because of the long half-life of vitamin D accumulation in tissues; excessive intake of vitamin D can cause chronic toxic effects, which present ashypercalcemia and renal damage.

Further controlled randomized trials on the effects of vitamin D supplementation are warranted to assess the relevance of vitamin D for liver fibrosis progression in HIV/HCV coinfected patients.

## 5. Conclusions

Our review indicates that vitamin D supplementation can be considered a relatively inexpensive therapeutic option to lower HCV-related fracture risk, owing to its benefic effect in reducing liver fibrosis progression in HIV/HCV coinfected patients. Other determinants of HCV-related increased fracture risk have still to be defined.

A good compromise between different opinions could be to start with relatively higher doses of vitamin D in HIV-HCV infected patients, skipping some steps of dose supplementation until more information is available to settle the question.

## What Is New?

*What Is Known?*

- Experimental evidence suggested a hepatoprotective role of vitamin D.
- Practitioners are often concerned about the lack of well-characterized data on the therapeutic value of Vitamin D supplementation to reduce liver fibrosis progression in HIV/HCV coinfected patients.

*What Is New?*

- Only particularly low levels of vitamin D should be considered in HIV/HCV coinfected patients.
- This systematic review reveals a recent interest in vitamin D supplementation as a relatively inexpensive therapeutic option to reduce HCV-related increased fracture risk by modifying liver fibrosis progression.

*What Does This Mean?*

- Among HIV-infected patients, the association between 25(OH)D levels and severity of liver disease partly explains the HCV-associated increased risk of osteoporotic fractures, while other determinants have still to be defined.
- Further RCTs are warranted to determine what level of vitamin D insufficiency and deficiency places an individual at risk of cirrhosis evolutions and what is the optimal dosage of vitamin D3 to exert sufficient antifibrosis effects in HIV/HCV coinfected populations.

## Figures and Tables

**Table 1:** Characteristics of the selected studies.

Study, year	Country	Total number	HIV+/HCV+ number	Control Group(s)	Age (yrs) HIV+/HCV+	Study design	Population study/setting	Topics
Mandorfer et al., 2015 [34]	USA	86	86		38.7 median	Cohort retrospective	HIV/HCV coinfected	Vitamin D levels. Other variables and severity of liver disease

Dong et al., 2014 [22]	USA					Systematic review and meta-analysis	HIV/HCV coinfected	Osteoporosis and fractures

Guzmán-Fulgencio et al., 2014 [31]	Spain	174	174	HIV/HCV coinfection	40.8 median	Cross-sectional	HIV/HCV coinfected	Prevalence of vitamin D levels and association with other parameters

Avihingsanon et al., 2014 [32]	Australia	331	130	Monoinfected HCV− HIV/HCV coinfection	42 median	Cross-sectional	HIV/HCV coinfected	Vitamin D levels and virological response in coinfection treatment

Luetkemeyer et al., 2013 [20]	USA					Review	monoinfected HIV and HIV/HCV coinfected	Bone metabolisms and vitamin D deficiency

Mandorfer et al., 2013 [35]	Austria	65	65	HIV/HCV coinfection	38.6 median	Cross-sectional	HIV/HCV coinfected	Vitamin D levels and virological response in coinfection treatment

Branch et al., 2013 [36]	USA	144	144	non-EVR^*^ HIV/HCV coinfected	48 median	Cohort retrospective	HIV/HCV coinfected genotype 1 treated in ACTG study	Vitamin D levels and virological response in coinfection treatment

El-Maouche et al., 2013 [33]	USA	116	116	HIV/HCV coinfection	49.9 median	Cohort prospective	HIV/HCV coinfected	Vitamin D levels and bone mineral density (BMD)

Linari et al., 2013 [37]	Italy	78	26	Monoinfected (HIV+)/Uninfected	45.8 mean	Cross-sectional	Haemophilia	Prevalence of hypovitaminosis D and BMD markers

Maalouf et al., 2013 [21]	USA	56.660	17.734	Monoinfected (HIV+)	44 median	Cohort retrospective	HIV-infected population	HCV-associated risk of osteoporotic fractures and severity of liver disease

Vecchi et al., 2012 [38]	Italy	120	41	Monoinfected (HIV+)/Uninfected	47 mean	Cohort prospective	HIV-infected population	Vitamin D levels and BMD; dairy calcium intake

Milazzo et al., 2011 [39]	Italy	237	93	Monoinfected HIV/Uninfected	45 median	Cross-sectional	HIV-infected population	Vitamin D levels and severity of liver disease

^*^Early virologic response.
